# Development and clinical feasibility study of a brief version of an addiction‐focused phenotyping battery in females receiving buprenorphine for opioid use disorder

**DOI:** 10.1002/brb3.3128

**Published:** 2023-06-27

**Authors:** Anna Beth Parlier‐Ahmad, Michelle Eglovitch, Catherine Legge, Lori A. Keyser‐Marcus, James M. Bjork, Amanda Adams, Tanya Ramey, Frederick Gerard Moeller, Caitlin E. Martin

**Affiliations:** ^1^ Department of Psychology Virginia Commonwealth University Richmond Virginia USA; ^2^ School of Medicine Virginia Commonwealth University Richmond Virginia USA; ^3^ Institute for Drug and Alcohol Studies Virginia Commonwealth University Richmond Virginia USA; ^4^ National Institute of Drug Abuse Bethesda Maryland USA; ^5^ Department of Obstetrics and Gynecology School of Medicine Virginia Commonwealth University Richmond Virginia USA

**Keywords:** buprenorphine, neurofunction, opioid use disorder, Phenotyping Assessment Battery (PhAB)

## Abstract

**Introduction:**

We aimed to streamline the NIDA Phenotyping Assessment Battery (PhAB), a package of self‐report scales and neurobehavioral tasks used in substance use disorder (SUD) clinical trials, for clinical administration ease. Tailoring the PhAB to shorten administration time for a treatment setting is critical to expanding its acceptability in SUD clinical trials. This study's primary objectives were to develop a brief version of PhAB (PhAB‐B) and assess its operational feasibility and acceptability in a female clinical treatment sample.

**Methods:**

Assessments of the original PhAB were evaluated along several criteria to identify a subset for the PhAB‐B. Non‐pregnant females (N=55) between ages 18–65, stabilized on buprenorphine for opioid use disorder (OUD) at an outpatient addiction clinic, completed this abbreviated battery remotely or after a provider visit in clinic. Participant satisfaction questions were administered. REDCap recorded the time to complete PhAB‐B measures.

**Results:**

The PhAB‐B included 11 measures that probed reward, cognition, negative emotionality, interoception, metacognition, and sleep. Participants who completed the PhAB‐B (N =55) were 36.1 ± 8.9 years of age, White (54.5%), Black (34.5%), and non‐Latinx (96.0%). Most participants completed the PhAB‐B remotely (*n* = 42, 76.4%). Some participants completed it in‐person (*n* = 13, 23.6%). PhAB‐B mean completion time was 23.0 ± 12.0 min. Participant experiences were positive, and 96% of whom reported that they would participate in the study again.

**Conclusion:**

Our findings support the clinical feasibility and acceptability of the PhAB‐B among a female opioid use disorder outpatient addiction treatment sample. Future studies should assess the PhAB‐B psychometric properties among broader treatment samples.

## Graphical abstract

This study's primary objectives were to develop a brief version of the NIDA Phenotyping Assessment Battery (PhAB) and assess its operational feasibility and acceptability in a female clinical treatment sample. Non‐pregnant females (N = 55) between ages 18–65, stabilized on buprenorphine for opioid use disorder (OUD) at an outpatient addiction clinic, completed this 11‐measure abbreviated battery (PhAB‐B). Findings support the clinical feasibility and acceptability of the PhAB‐B among a female OUD outpatient addiction treatment sample.

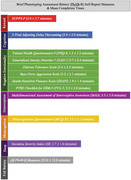

## 1 INTRODUCTION

Addiction represents an important public health concern as life expectancy in the United States continues to decrease, largely due to increased substance use‐related deaths like overdose (Woolf & Schoomaker, 2019). Evidence‐based addiction treatments, both pharmacological and behavioral, are effective (Witkiewitz et al., 2019). However, most people with substance use disorder (SUD) do not receive treatment (Martin et al., 2021). Among those who do, treatment attrition and substance use recurrence are common (McLellan et al., 2000). Strategies to expand and personalize SUD treatment options using precision medicine principles are needed.

Precision medicine entails tailoring treatment regimens based on individual characteristics to improve outcomes (Volkow, 2020). SUD treatment provides an opportunity for advances in precision medicine because people with SUD comprise an extremely heterogeneous group in terms of their biological, psychosocial, and neurobehavioral characteristics and related drivers of substance use (Leggio et al., 2009; Litten et al., 2015). For example, according to the Diagnostic and Statistical Manual of Mental Disorders‐5, over 2000 different symptom combinations would all meet the criteria for alcohol use disorder (AUD) (APA, 2013; Witkiewitz et al., 2019). Current diagnostic and treatment practices do not usually account for these individual characteristics which limits treatment efficacy and contributes to heterogeneity in SUD treatment response (Huhn et al., 2019; Panlilio et al., 2019; Saraiya et al., 2020).

The utility of deep phenotyping of individuals with SUD to characterize the function of specific neurofunction domains has received attention as a strategy to better address heterogeneity in addiction (Keyser‐Marcus et al., 2021; Kwako et al., 2016). The Addictions Neuroclinical Assessment (ANA) battery was developed by the National Institute on Alcohol Abuse and Alcoholism for use in AUD clinical trials to probe negative emotionality, incentive salience, and executive function as domains considered critical to AUD (Kwako et al., 2019). However, a comprehensive ANA battery takes approximately 10 hours to complete across 2 days of administration (Kwako et al., 2016), limiting its feasibility in a clinical trial and addiction treatment setting.

To account for a broader range of neurofunctional domains involved in the addiction process, the National Institute for Drug Abuse (NIDA) subsequently developed the Phenotyping Assessment Battery (PhAB), which expanded upon the three‐domain ANA model to also include interoception, metacognition, and sleep domains. The PhAB is a comprehensive package of self‐report psychometric scales and neurocognitive behavioral tasks, with an optional resting state fMRI, designed for deployment in any type of SUD clinical trial (Keyser‐Marcus et al., 2021). A prior PhAB feasibility study conducted among a sample of individuals with and without opioid, stimulant, and/or cannabis use disorders found the PhAB to be feasible in a research laboratory setting with substantially lower participant burden than the ANA battery (<2‐hour completion time) (Keyser‐Marcus et al., 2021). The PhAB was also rated as highly acceptable to participants. However, for prospective studies to be conducted within outpatient SUD treatment clinics, investigators may find the practicality of even the 2‐hour in‐person PhAB assessment to be unfeasible. Recruiting SUD treatment patients to complete study assessments can be challenging, especially if the assessments are lengthy, necessitate in‐person completion, or require a separate visit to a research testing facility.

Therefore, while the NIDA PhAB is a promising deep phenotyping battery, its acceptance into clinical research practice is slow, driven in part by the impracticality of administering an extensive battery in a fast‐paced clinic (Keyser‐Marcus et al., 2021). A shortened version of the PhAB to optimize administration in a realistic SUD treatment clinical setting is needed. Additionally, sex is an important variable that modifies SUD trajectories (Huhn et al., 2019). A first step to developing a sex‐informed phenotyping tool is to stratify samples by sex when assessing abbreviated PhAB options. Females have been largely underrepresented in phenotyping studies among SUD clinical samples, warranting their priority in these initial investigations (Kwako et al., 2019; Nieto et al., 2021). The current study's objectives were to (1) describe the development of a brief version of the PhAB (PhAB‐B), (2) assess the feasibility of the PhAB‐B in a treatment setting, and (3) evaluate the acceptability of the PhAB‐B among a female outpatient treatment sample receiving buprenorphine for opioid use disorder (OUD).

## 2 METHODS

### 2.1 Development of the PhAB‐B

A multidisciplinary workgroup (N = 7) of clinician (CEM and FGM) and non‐clinician addiction researchers (LKM, JMB, and TR) and research assistants (AA and MM) convened with the goal to streamline the PhAB into an abbreviated battery, PhAB‐B, and optimize its suitability for an outpatient SUD treatment clinical setting, while also retaining phenotypic coverage of all domains in the original PhAB conceptual framework. Ongoing COVID‐19 concerns, coupled with the superior test‐retest reliability of trait‐like questionnaire probes of day‐to‐day temperament and other phenotypes relative to that of neurocognitive performance task scores, prompted an emphasis on self‐report instruments that could be administered remotely (Enkavi et al., 2019). To inform their review, the workgroup used data from the original PhAB feasibility and acceptability study, including measure completion times and observations by the study team administering the PhAB (Keyser‐Marcus et al., 2021).

Each workgroup meeting (*n* = 10) focused on a PhAB domain. If a member of the workgroup was not able to be present at the meeting, their input was followed up via email or a separate meeting with the workgroup leader (CEM). At least one clinician, one non‐clinician researcher, and one research assistant were present at every workgroup meeting. Together, the workgroup qualitatively weighed the pros and cons of including each original PhAB measure for feasibility and acceptability in an SUD treatment setting while also balancing the measure's contribution to the robustness of the phenotyping battery (Table 1). Workgroup discussions continued until a consensus was reached on decisions for included PhAB‐B measures. For example, in the reward domain, the Short UPPS‐P Impulsive Behavior Scale from the original PhAB was selected for inclusion in the PhAB‐B, given its brevity, ability to be self‐completed remotely, and its acceptance as a valuable global measure of the reward domain (Cyders et al., 2014). Conversely, research assistant representatives expressed concern about the feasibility of the Line Counting task explaining that many SUD participants in prior studies required considerable staff assistance and time to complete it, making it unfeasible to administer quickly in a fast‐paced clinical environment or self‐complete remotely; thus, it was excluded from the PhAB‐B.

**TABLE 1:** Summary of revisions to original PhAB during the development of PhAB‐B. PhAB‐B, a brief version of the NIDA Phenotyping Assessment Battery.

Original PhAB measures	Revision	Disadvantage(s) of original PhAB measure	Advantage(s) of PhAB‐B measure	PhAB‐B measures
Reward domain				
SUPPS‐P: Short Impulsive Behavior Scale	Retain	–	‐ Brief version preserves psychometric properties ‐ Global measure of reward ‐ Ease of remote administration	SUPPS‐P: Short Impulsive Behavior Scale
Line Counting Task	Exclude	‐ Administration challenges, especially older participants ‐ Remote option challenging for SUD patients	–	–
Hypothetical purchase task	Exclude	‐ Challenging to complete for participants engaged in SUD treatment and not actively using non‐prescribed substances	–	–
Cognition domain				
Five‐Trial Delay Discounting	Retain	–	‐ Associated with SUD treatment outcomes in the existing literature ‐ Ease of remote administration	Five‐Trial Delay Discounting
Backwards Digit Span	Exclude	‐ Remote option challenging for SUD patients	‐ Brief, acceptable to participants	Optional use as a supplemental measure. If feasible to administer remotely could be included in PhAB‐B
Attentional Network Task	Exclude	‐Administration challenges ‐ Remote option challenging for SUD patients	‐ Valuable measure of attentional and orienting function	Optional use as a supplemental measure. If feasible to administer remotely could be included in PhAB‐B
Stop Signal Task	Exclude	‐ Administration challenges ‐ Remote option challenging for SUD patients	–	–
Negative emotionality				
Emotional Go‐No Go	Exclude	‐ Administration challenges ‐ Remote option challenging for SUD patients	–	–
PROMIS‐Depression	Alternative measure	‐Not clinically applicable ‐ Norms based on general population	‐ Psychometrically sound measure of depressive symptoms ‐Brief ‐ Clinically relevant for an SUD treatment setting	PHQ‐9: Patient Health Questionnaire
PROMIS‐Anxiety	Alternative measure	‐Not clinically applicable ‐ Norms based on general population	‐ Psychometrically sound measure of anxiety symptoms ‐ Brief ‐ Clinically relevant for an SUD treatment setting	GAD‐7: Generalized Anxiety Disorder
DTS: Distress Tolerance Scale	Retain	‐	‐ Brief ‐ Ease of remote administration	DTS: Distress Tolerance Scale
Buss Perry Aggression Questionnaire	Retain	–	‐ Widely used, comprehensive measure of aggression ‐ Participants can complete quickly	Buss Perry Aggression Scale
SHAPS: Snaith Hamilton Pleasure Scale	Retain	‐ The wording of some questions confusing to participants	‐ Brief ‐ Widely used measure of anhedonia ‐ Ease of remote administration	SHAPS: Snaith Hamilton Pleasure Scale
	Addition (from original PhAB platform measures)	‐ PTSD measure was not included in the original PhAB core phenotyping measures	‐ Brief and clinically relevant for an SUD treatment setting	PCL‐5: PTSD Checklist
Interoception				
MAIA: Multidimensional Assessment of Interoceptive Awareness	Retain	–	‐ Appropriate measure of interoception ‐ Acceptable to participants ‐ Ease of administration remotely	MAIA: Multidimensional Assessment of Interoceptive Awareness
Metacognition				
MCQ‐30: Metacognitions Questionnaire	Retain	–	‐ Appropriate measure of metacognition ‐ Acceptable to participants ‐ Ease of administration remotely	MCQ‐30: Metacognitions Questionnaire
Sleep				
PSQI: Pittsburgh Sleep Quality Index	Alternative measure	‐ Lengthy ‐ Remote administration challenges (e.g., fill in the blank items, missing data) ‐ Complex scoring	‐ Insomnia is the most common sleep condition in SUD treatment populations ‐Brief ‐ Clinically relevant for an SUD treatment setting	ISI: Insomnia Severity Index

Abbreviations: PTSD, post‐traumatic stress disorder; SUD, substance use disorder.

The workgroup discussed if any NIH Common Data Elements (CDE) not in the original PhAB should be added to ensure a comprehensive assessment of the assigned domain. The NIH CDE are defined variables intended for use across studies to standardize data collection (NIH, 2023). For example, in the negative emotionality domain, the workgroup preserved three original PhAB phenotyping measures in the PhAB‐B (Buss & Perry, 1992; Simons & Gaher, 2005; Snaith et al., 1995) and replaced the Patient‐Reported Outcomes Measurement Information System (PROMIS) Depression and Anxiety scales in the PhAB with alternate measures commonly used in clinical practice, the Patient Health Questionnaire‐9, and General Anxiety Disorder‐7 (Table 1) (Kroenke et al., 2001; Measures, 2020; Spitzer et al., 1960). A post‐traumatic stress disorder (PTSD) symptom measure (PCL‐5 PTSD Checklist) was added given the high prevalence of PTSD and comorbidity with other mental health conditions common in the SUD treatment population (Blevins et al., 2015).

### 2.2 Pilot testing the PhAB‐B

#### 2.2.1 Participants and study design

The pilot study of the PhAB‐B battery was a cross‐sectional survey nested within a larger study assessing the prevalence and correlates of insomnia among individuals in outpatient OUD treatment with buprenorphine. The parent study intentionally focused on recruiting a convenience sample of female participants, given the known higher burden of insomnia among women than men (Suh et al., 2018). Inclusion criteria were age between 18 and 65 years, female sex, not currently pregnant or within six weeks of pregnancy, having an OUD diagnosis, receiving buprenorphine for OUD for at least 6 weeks, and speaking English.

Potential participants were approached in the outpatient SUD treatment clinic between February and November 2022. All participants provided informed consent. After consent, participants completed a brief research assistant interview (e.g., medical history, current medications, and substance use) and then were offered two options for completion of the parent study's electronic survey: (1) in‐person on a tablet provided in the outpatient clinic or (2) remotely on their own smart device via a secure REDCap link. Participants were encouraged to complete the surveys as soon as possible, within a maximum of 3 weeks of the interview, and were allowed to start/stop the surveys at their convenience. The PhAB‐B battery was embedded at the end of the parent study survey. A medical record review was conducted to abstract clinically relevant variables (i.e., insurance and length of time receiving buprenorphine). Participants were compensated $15 for the completion of the parent study. This study was approved by the University's Institutional Review Board (#HM200233390).

#### 2.2.2 SUD treatment clinical setting

The outpatient SUD treatment clinic is affiliated with a safety net academic medical center in a Medicaid‐expanded southern state. The clinic provides outpatient SUD treatment for over 500 adults yearly, predominately low‐income, racially, and ethnically minoritized people, with approximately 90% receiving buprenorphine for OUD. Most patients are referred from within the academic medical center (e.g., inpatient consults and primary care physicians). A comprehensive, recovery‐oriented care model is utilized throughout treatment in which patients have access to medical, psychiatric, mental health, case management, and social work services. The clinic prioritizes a low‐threshold, harm reduction approach, meaning that established patients with substance use recurrence are not excluded from treatment, but instead provided with increased wrap‐around support. Additionally, there is no time limitation on how long patients can continue SUD treatment with the clinic. Therefore, the patient population in the clinical study setting represents many stages across the OUD treatment and recovery cascade.

#### 2.2.3 PhAB‐B survey measures

Within the reward domain, the 20‐item short version of the UPPS‐P (SUPPS‐P) Impulsive Behavior Scale assessed facets of impulsivity across five subscales (urgency, premeditation, perseverance, sensation seeking, and positive urgency) (Cyders et al., 2014).

Within the cognition domain, the five‐trial delay discounting task assessed an individual's discount rate by presenting a series of questions in which the participant chooses between some amount of a delayed commodity or an amount available immediately (Koffarnus & Bickel, 2014). The brief task is based on the premise that individuals tend to value rewards less as the amount of time increases until those rewards would be received.

Within the negative emotionality domain, the nine‐item PHQ‐9 assessed the frequency of depressive symptoms over the past 2 weeks (Kroenke et al., 2001). One item on the PHQ‐9 assesses the frequency of suicidal ideation. If a participant endorsed suicidality (*n* = 15), the study research assistant informed the principal investigator and contacted the participant within 24 hours to conduct a risk assessment. If the research assistant was unable to reach the participant after three attempts, she contacted the emergency contact listed in the medical record. The seven‐item Generalized Anxiety Disorder‐7 (GAD‐7) assessed the frequency of core symptoms of GAD over the past 2 weeks (Spitzer et al., 1960). The 15‐item Distress Tolerance Scale measured emotional distress tolerance across four factors (tolerance, absorption, appraisal, and regulation) (Simons & Gaher, 2005). The 29‐item Buss‐Perry Aggression Scale assessed aggression across four factors (physical aggression, verbal aggression, anger, and hostility) (Buss & Perry, 1992). The 14‐item Snaith Hamilton Pleasure Scale measured four domains of pleasure response (e.g., anhedonia) over the past few days (Snaith et al., 1995). The 20‐item Posttraumatic Stress Disorder Checklist corresponding to the DSM‐5 (PCL‐5) measured symptoms of PTSD during the past month (Blevins et al., 2015).

Within the interoception domain, the 32‐item Multidimensional Assessment of Interoceptive Awareness (MAIA) measured interoceptive body awareness across five dimensions, including emotional/physiological state, physical discomfort, and pain (Mehling et al., 2012).

Within the metacognition domain, the 30‐item Metacognitions Questionnaire (MCQ‐30) assessed metacognitive beliefs across five subscales relevant to psychopathology, including cognitive confidence, positive beliefs about worry, cognitive self‐consciousness, negative beliefs about the uncontrollability of thoughts and danger, and beliefs about the need to control thoughts (Wells & Cartwright‐Hatton, 2004).

Within the sleep domain, the seven‐item Insomnia Severity Index assessed the perceived severity of insomnia symptoms during the past 2 weeks (Bastien et al., 2001; Morin et al., 2011).

#### 2.2.4 Feasibility and tolerability metrics

The primary feasibility outcomes assessed the ease of PhAB‐B administration and participation burden in a clinical SUD treatment setting. Feasibility outcomes were operationalized as individual measure completion times and PhAB‐B total completion time, both measured by REDCap, as well as the type of study visit (in‐person or remote). Additionally, we assessed representativeness of those who completed the PhAB‐B battery (PhAB‐B completers).

Secondarily, the acceptability of the PhAB‐B was assessed by participant satisfaction with the overall study (i.e., “I would participate in this study again” and “I would recommend this study to family and friends”) upon completion of the PhAB‐B measures in the survey.

#### 2.2.5 Additional descriptive measures

Recent non‐prescribed drug and alcohol use were assessed in the interview using the Timeline Follow Back, a calendar method used to collect daily alcohol and non‐prescribed drug use data over the past 28 days (Sobell & Sobell, 1992). For those who did not complete the interview (*n* = 9), recent non‐alcohol substance use was assessed by a review of recent urine drug test results (within the past 28 days) documented in the medical record. Age, race, and ethnicity were self‐reported in the parent study's survey. For participants who consented to the parent study but did not complete any other parent study procedures (*n* = 9) or did not initiate the parent study survey (*n* = 27), these variables were abstracted from the medical record. For all participants, insurance status and length of time receiving buprenorphine were abstracted from the medical record. The length of time receiving buprenorphine was operationalized as the number of months between the buprenorphine induction date and the study enrollment date.

#### 2.2.6 Data analyses

PhAB‐B measures within each PhAB domain were summarized. Descriptive statistics were generated for feasibility and acceptability data for the pilot sample of PhAB‐B completers. Key sociodemographic and clinical characteristics were compared between PhAB‐B completers and PhAB‐B non‐completers using chi‐square or Fisher's exact test for categorical variables and *T*‐tests and Mann Whitney *U* for continuous variables. PhAB‐B completers were defined as participants who completed the PhAB‐B battery nested within the parent study's survey. PhAB‐B non‐completers were defined as any participant who consented to study participation but did not complete the PhAB‐B battery. Significance was set at .05. Analyses were performed using IBM SPSS Statistics 27.0.

## 3 RESULTS

### 3.1 Development of the PhAB‐B

The PhAB‐B includes a total of 11 self‐report measures across the six addiction‐relevant neurofunctional domains in the original PhAB. Table 1 details the rationale for PhAB‐B measure inclusion/exclusion from the PhAB as determined by the workgroup. All measures are available through REDCap and can be completed remotely.

### 3.2 Pilot testing the PhAB‐B

**FIGURE 1:**
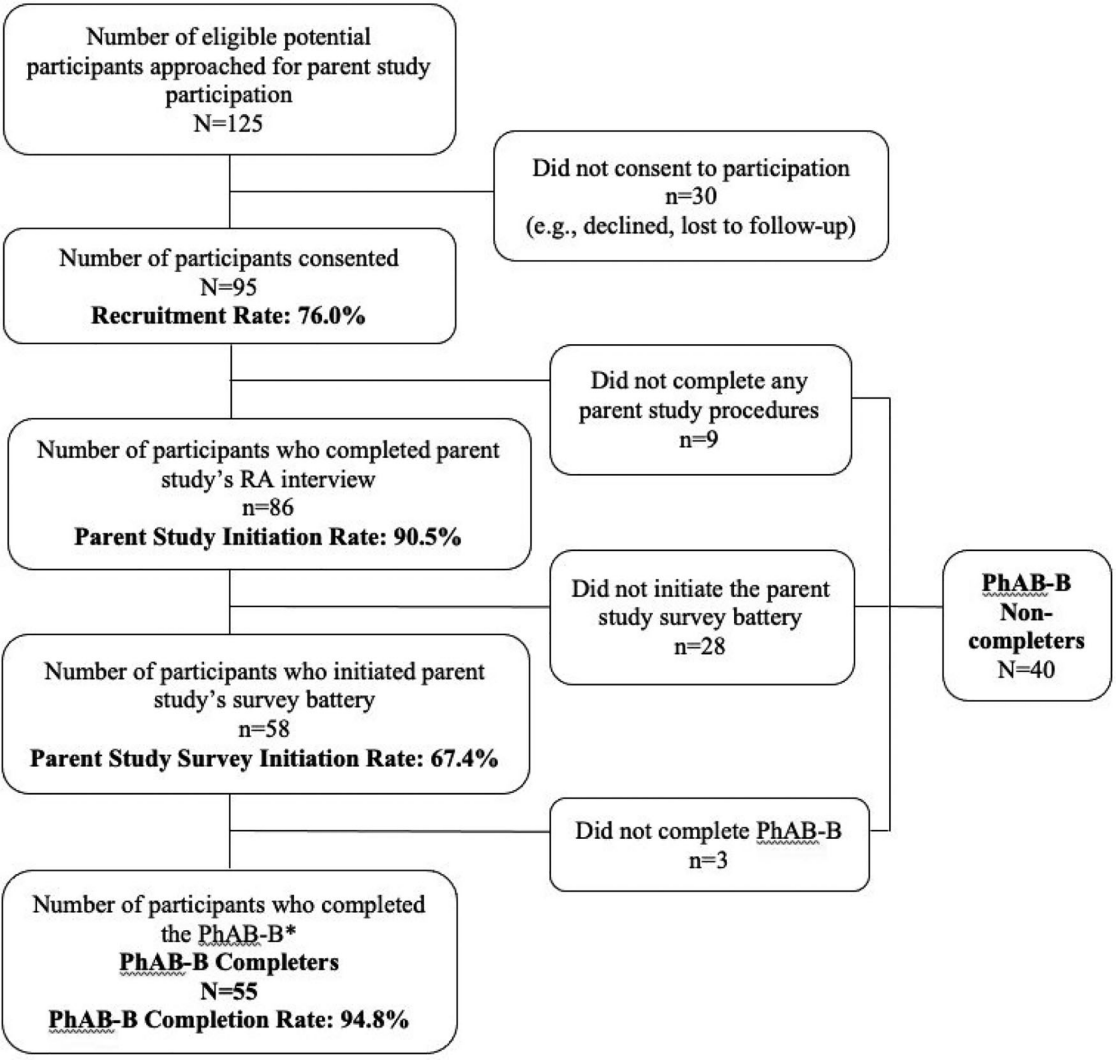
Flow diagram of study participation among the pilot sample of female participants stabilized on buprenorphine for opioid use disorder (OUD) from parent study recruitment through PhAB‐B battery completion. *Note*: *PhAB‐B battery was embedded at the end of the parent study's survey. PhAB‐B, a brief version of the NIDA Phenotyping Assessment Battery.

As shown in the participant flow diagram (Figure 1), N = 95 female patients consented to parent study participation (76.0% recruitment rate). Of those, *n* = 40 (42.2%) participants were lost to follow‐up between study enrollment and initiating the PhAB‐B battery (PhAB‐B non‐completers). All participants who initiated the PhAB‐B battery (*n* = 55, PhAB‐B completers) completed the full battery (100% PhAB‐B completion rate) indicating the battery was tolerable to all of those who started it during pilot testing. PhAB‐B completers and non‐completers did not differ on demographic variables or recent substance use (Table 2). PhAB‐B completers had been receiving buprenorphine for significantly shorter time than PhAB‐B non‐completers (*p* = .009).

**TABLE 2:** Sociodemographic and clinical characteristics among the pilot sample of female participants stabilized on buprenorphine for opioid use disorder (OUD) who completed the PhAB‐B battery (N = 55) and those who did not (N = 40).

Characteristic	PhAB‐B completers N = 55 N (%)	PhAB‐B non‐completers N = 40 N (%)	*p*‐Value
Age (mean ± SD, years)	36.1 ± 8.9	34.6 ± 7.6	.619
Race			
American Indian/Alaska Native	2 (3.6)	0 (0)	.171
Black	19 (34.5)	18 (45.0)	
White	30 (54.5)	22 (55.0)	
More than one race	4 (7.3)	0 (0)	
Ethnicity			
Hispanic/Latinx	2 (3.6)	0 (0)	.306
Non‐Hispanic/Latinx	48 (87.3)	40 (100)	
Not reported	5 (9.1)	0 (0)	
Insurance			
Public	52 (94.5)	36 (90.0)	.326
Private	3 (5.5)	4 (10.0)	
Length of time receiving buprenorphine [median (range), months]	21.9 (1.9–61.2)	30.5 (4.6–63.4)	.009
Recent non‐prescribed substance use (past 28 days)			
Any non‐prescribed substance use	31 (56.4)	24 (60.0)	.616
Opioid	0 (0)	3 (7.5)	
Stimulant	8 (14.5)	7 (17.5)	
Cannabis	23 (41.8)	17 (42.5)	
Sedative	1 (1.8)	2 (5.0)	
Alcohol	10 (18.2)	10 (25.0)	

Among the PhAB‐B pilot completers (N = 55), participants were 36.1 ± 8.9 years of age and predominately White (54.5%), Black (34.5%), and non‐Latinx (87.3%) (Table 2). Nearly all were publicly insured. Median length of time receiving buprenorphine was almost 1 year. Over half self‐reported recent non‐prescribed substance use, most commonly cannabis use. Of participants who endorsed suicidality on the PHQ‐9 (*n* = 15), the research assistant completed risk assessments with seven. Based on medical record review, all 15 participants remained engaged in OUD treatment with regular provider interactions where suicidality and safety assessments are routine.

**FIGURE 2:**
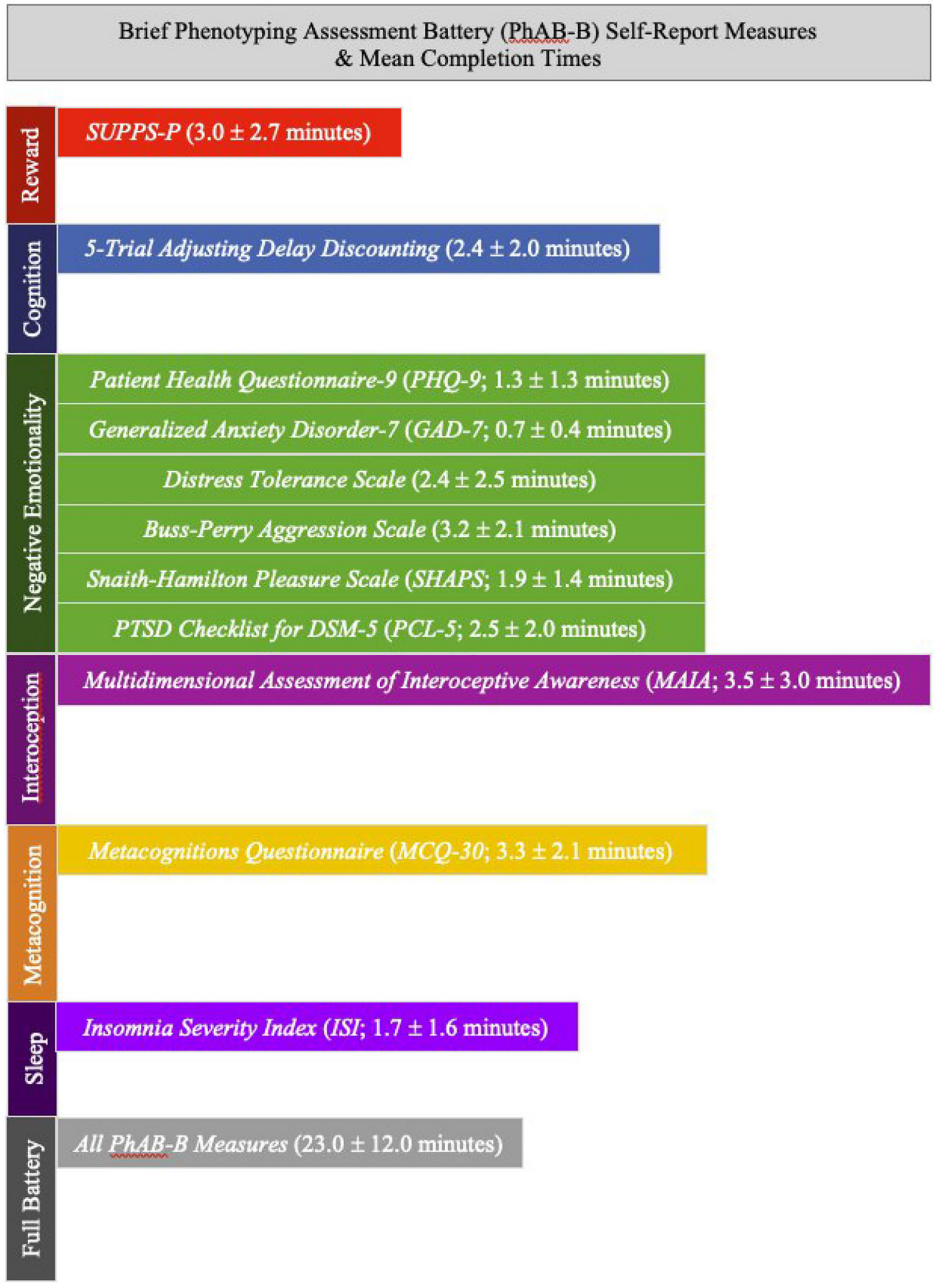
PhAB‐B measures within the Phenotyping Assessment Battery (PhAB) neurofunctional domains as well as mean individual measure and PhAB‐B battery total completion times among the pilot sample of female participants stabilized on buprenorphine for opioid use disorder (OUD) (N = 55). PhAB‐B, a brief version of the NIDA Phenotyping Assessment Battery.

Three‐fourths of participants who completed the battery did so remotely (*n* = 42, 76.4%). The remaining one‐fourth (*n* = 13, 23.6%) completed it in‐person. Individual measure completion time took under 4 min each on average (Figure 2). The lengthiest measures were the MAIA (interoception domain) and the MCQ‐30 (metacognition domain). The shortest measures were the PHQ‐9 and GAD‐7 (negative emotionality domain). The PhAB‐B total mean completion time was 23.0 ± 12.0 min.

Participant experiences were positive, with 96.4% (*n* = 53) endorsing that they would participate in the study again, and 92.7% (*n* = 51) reporting that they would recommend the study to their family and friends.

## 4 DISCUSSION

To advance the use of precision medicine principles in addiction treatment, assessments that identify psychobiologically meaningful individual differences with sufficient brevity to be acceptable to patients and research participants are essential. (Volkow, 2018) The current study sought to describe the development of the PhAB‐B as well as assess the feasibility and acceptability of the PhAB‐B in a clinical research setting. Among an outpatient sample of female patients receiving buprenorphine, we found that the PhAB‐B was feasible to administer either remotely or in‐person in this treatment setting, and it was not considered burdensome to participants. Notably, the sample largely consisted of patients identifying with marginalized groups in a safety‐net care system at various stages of the OUD treatment and recovery cascade.

Our pilot study intentionally took place in a realistic clinical research setting. Generally, outpatient SUD treatment clinics are fast‐paced, clinically‐focused environments with competing demands on clinicians and patients. Clinicians typically have 15‐to‐20‐min appointments with 20–30 patients per day, during which they have many responsibilities (e.g., reconciling medications, reviewing SUD status, and performing a physical exam). Additionally, patients with SUD receiving care in an outpatient setting often have chaotic lives and additional challenges including management of medical comorbidities, psychosocial stressors, child care, and interpersonal challenges (Parlier‐Ahmad et al., 2021). Therefore, time and space are often significant barriers to conducting research in this setting. Because clinical care is the top priority, research must fit seamlessly into the clinical flow. Our intentionality to minimize participant burden in the adaptation of the PhAB‐B likely contributed to its high acceptability ratings. Future studies evaluating the PhAB‐B and other phenotyping batteries for people with addiction should prioritize research in realistic settings, such as SUD treatment clinics located in both rural and urban locations.

Even when research studies are designed to be low‐burden, it can still be challenging to recruit and retain participants from an outpatient SUD treatment setting (Fallin‐Bennett et al., 2022). We observed a relatively high recruitment rate (76%) and a high PhAB‐B completion rate (100%) among those who initiated the battery (higher than the 83% completion rate in the original PhAB feasibility study). However, there was a substantial group of participants who were lost to follow‐up between study enrollment and PhAB‐B initiation (42%). In the original PhAB feasibility study, participants with SUD completed study procedures in a clinical research laboratory as part of a dedicated visit with known expectations of research procedures (Keyser‐Marcus et al., 2021). In contrast, participation in this pilot was ancillary to a clinical care visit, and participants were invited to complete the battery as part of a larger study.

PhAB‐B completers were broadly similar to the population of female patients in outpatient OUD treatment. The length of time receiving buprenorphine was the only group difference identified between PhAB completers and non‐completers. PhAB‐B completion rates may be higher among participants who had been receiving buprenorphine for a shorter duration of time because patients tend to have more frequent interfacing with the clinic (and research team members) at treatment onset. However, in both the PhAB‐B completer and non‐completer groups, there was a wide range in treatment length underscoring the many stages across the OUD treatment and recovery cascade represented in this sample. Among PhAB‐B completers, feasibility was supported, and acceptability ratings were overwhelmingly positive. Even though we had positive findings among our PhAB‐B completers, future studies need to assess how to make all aspects of clinical research more feasible and acceptable to diverse SUD treatment populations (Fallin‐Bennett et al., 2022).

We hypothesize that the primary factors that positively contributed to the feasibility and acceptability of the PhAB‐B in this outpatient SUD treatment setting were the ability to complete the entire battery remotely and its brevity. During the COVID‐19 pandemic, telehealth‐delivered addiction care was adopted by many SUD treatment centers and has persisted as an important mode of healthcare delivery in outpatient SUD treatment (Mark et al., 2022). In line with this paradigm shift in clinical care, providing both remote and in‐person options for clinical research participation should be prioritized in the post‐COVID era. In our pilot study, three in four participants opted to complete the PhAB‐B measures remotely. While we did not compare in‐person to remote completers due to the small sample size, our findings underscore the importance of offering flexible study completion options. Future work is needed comparing the validity of addiction phenotyping neurofunctional measures by administration method (virtual vs. in‐person) and setting (in‐home vs. in‐clinic vs. in‐laboratory). Additionally, as the PhAB‐B is adapted for other studies, it is notable that contacting participants remotely can be challenging (e.g., <50% reached for PHQ‐9 risk assessment); this is an important consideration, particularly if the PhAB‐B is going to be employed with people not engaged in regular SUD treatment. Markedly, the PhAB‐B average total completion time was 23 min, over an hour shorter than the original PhAB (Keyser‐Marcus et al., 2021). This reduced time burden reflects how the PhAB‐B is a promising phenotyping option to consider for integration into future research studies, as participants can feasibly complete the PhAB‐B measures in conjunction with a clinic visit or during their busy lives outside of the clinic. Finally, we note that questionnaire metrics tend to have better test‐retest reliability than cognitive performance assessments, especially as they may relate to inhibitory and other executive functions (Enkavi et al., 2019).

The current pilot study was performed at a single clinic; therefore, the generalizability of results is limited. Similarly, the acceptability of the PhAB‐B may be lower in non‐compensated clinical treatment settings, due to institutional mistrust among minoritized populations. The current feasibility study was nested within another study, likely contributing to participant fatigue and lost to follow‐up prior to PhAB‐B initiation. Given the small female‐only sample and low overall study completion rate, these findings are preliminary and intended to inform future research that can build upon this study by piloting the PhAB‐B among broader treatment samples, in terms of SUD type, sex, and gender, and assessing its psychometric properties.

## 5 CONCLUSIONS

This study is the first to describe the development and pilot testing of a brief version of the original PhAB tailored to optimize administration in an outpatient SUD treatment setting. A multidisciplinary group adapted the NIDA PhAB into a briefer version of the battery, PhAB‐B. Preliminary pilot testing findings support the clinical feasibility and acceptability of the PhAB‐B among a female outpatient OUD treatment sample. Unique operational improvements in the PhAB‐B such as the remote administration ability and focus on brevity may help overcome the barriers to conducting research in outpatient addiction treatment clinical settings. Additional research is needed to assess the generalizability and psychometric properties of PhAB‐B among larger, more heterogenous SUD treatment samples and by remote versus in person completion locations.

## CONFLICT OF INTEREST STATEMENT

Tanya Ramey was substantially involved in UG1DA050207 and U54DA038999, consistent with her role as Scientific Officer. She had no involvement in other cited grants. All authors declare no other relevant conflict of interest. The authors alone are responsible for the content and writing of this paper. Any opinion, findings, and conclusions or recommendations expressed in this material are those of the authors and do not necessarily reflect the views of the National Institute on Drug Abuse.

### PEER REVIEW

The peer review history for this article is available at https://publons.com/publon/10.1002/brb3.3128.

## Data Availability

Research data are not shared.
